# Effects of *Leea indica* leaf extracts and its phytoconstituents on natural killer cell-mediated cytotoxicity in human ovarian cancer

**DOI:** 10.1186/s12906-023-03904-1

**Published:** 2023-03-11

**Authors:** Soek-Ying Neo, Yin-Yin Siew, Hui-Chuing Yew, Yaqian He, Keng-Ling Poh, Yi-Chen Tsai, Shu-Ling Ng, Wei-Xun Tan, Teck-Ian Chong, Claire Sophie En-Shen Lim, Samuel Shan-Wei Ho, Deepika Singh, Azhar Ali, Yeh-Ching Linn, Chay-Hoon Tan, See-Voon Seow, Hwee-Ling Koh

**Affiliations:** 1 Department of Pharmacy, Faculty of Science, National University of Singapore, 18 Science Drive 4, Singapore, 117543 Singapore; 2 Cancer Science Institute of Singapore, 14 Medical Drive, Singapore, 117599 Singapore; 3 Department of Haematology, Singapore General Hospital, 20 College Road, Singapore, 169856 Singapore; 4 Department of Pharmacology, Yong Loo Lin School of Medicine, National University of Singapore, 16 Medical Drive, Singapore, 117600 Singapore; 5 National Cancer Centre Singapore, 11 Hospital Crescent, Singapore, 169610 Singapore

**Keywords:** *Leea indica*, Methyl gallate, NK cell, Oxaliplatin, Ovarian cancer

## Abstract

**Background:**

The rich biodiversity of medicinal plants and their importance as sources of novel therapeutics and lead compounds warrant further research. Despite advances in debulking surgery and chemotherapy, the risks of recurrence of ovarian cancer and resistance to therapy are significant and the clinical outcomes of ovarian cancer remain poor or even incurable.

**Objective:**

This study aims to investigate the effects of leaf extracts from a medicinal plant *Leea indica* and its selected phytoconstituents on human ovarian cancer cells and in combination with oxaliplatin and natural killer (NK) cells.

**Methods:**

Fresh, healthy leaves of *L. indica* were harvested and extracted in 70% methanol by maceration. The crude extract was partitioned with *n*-hexane, dichloromethane and ethyl acetate. Selected extracts and compounds were analyzed for their effects on cell viability of human ovarian cancer cells, NK cell cytotoxicity, and stress ligands expression for NK cell receptors. They were also evaluated for their effects on TNF-α and IL-1β production by enzyme-linked immunosorbent assay in lipopolysaccharide-stimulated human U937 macrophages.

**Results:**

Leaf extracts of *L. indica* increased the susceptibility of human ovarian tumor cells to NK cell-mediated cytotoxicity. Treatment of cancer cells with methyl gallate but not gallic acid upregulated the expression of stress ligands. Tumor cells pretreated with combination of methyl gallate and low concentration of oxaliplatin displayed increased levels of stress ligands expression and concomitantly enhanced susceptibility to NK cell-mediated cytolysis. Further, NK cells completely abrogated the growth of methyl gallate-pretreated ovarian cancer cells. The leaf extracts suppressed TNF-α and IL-1β production in human U937 macrophages. Methyl gallate was more potent than gallic acid in down-regulating these cytokine levels.

**Conclusions:**

We demonstrated for the first time that leaf extracts of *L. indica* and its phytoconstituent methyl gallate enhanced the susceptibility of ovarian tumor cells to NK cell cytolysis. These results suggest that the combined effect of methyl gallate, oxaliplatin and NK cells in ovarian cancer cells warrants further investigation, for example for refractory ovarian cancer. Our work is a step towards better scientific understanding of the traditional anticancer use of *L. indica.*

**Supplementary Information:**

The online version contains supplementary material available at 10.1186/s12906-023-03904-1.

## Introduction

Ovarian cancer remains the most lethal gynecological cancer among women [1, 2]. Projection estimates by GLOBOCAN 2020 indicated that by 2040, the number of women worldwide diagnosed with ovarian cancer will increase about 37% to 428,966 [3]. Further, the number of deaths from the disease is projected to surge over 50% to 313,617 from 2020. Current standard treatment for the most common ovarian cancer (i.e. epithelial ovarian cancer) includes surgery followed by platinum-based chemotherapy and radiation therapy [4]. The five-year survival rate of ovarian cancer is around 47%, mainly due to high risk of relapse and resistance to chemotherapy. Moreover, early-stage detection of the disease is difficult due to lack of promising screening tools, and most patients are typically diagnosed at advanced stage of the cancer. New therapeutic methods have emerged from various biomarker-driven initiatives such as poly ADP-ribose polymerase inhibitors and antiangiogenic therapy [5, 6]. Cancer immunotherapy, which is the modulation of the body’s innate immune system to treat cancer, has gained widespread interest as any immune-related adverse effects are relatively better tolerated than traditional chemotherapeutic agents [7]. Current immunotherapies for ovarian cancer fall into five broad categories: monoclonal antibodies, checkpoint inhibitors and immune modulators, therapeutic vaccines, adoptive T cell transfer and oncolytic viruses [4]. In particular, natural killer (NK)-cell based immunotherapy holds great promise for cancer treatment because NK cells can be easily isolated and expanded ex vivo for adoptive cell transfer therapy [8–10]. NK cells recognize a broad panel of several dozen ligands which can each induce a cytolytic response [10]. The advantage of NK cell-based therapy over T cells is that there is virtually no complication from graft-versus-host disease [8, 9]. There is no good treatment for late stage ovarian cancer after relapse from the treatment of bevacizumab and olaparib [5, 6]. NK cell therapy of relapsed cancer could potentially provide an alternative option [8–10].

Majority of the chemotherapeutic agents exert their cytotoxic effects by apoptosis which is typically deemed to be non-inflammatory and non-immunogenic [11]. However, it is now clear that certain agents such as anthracyclines and oxaliplatin, in addition to having cytotoxic properties, can also elicit immunogenic cell death [12]. Immunogenic cell death is mediated largely by damage-associated molecular patterns (DAMPs), most of which are recognized by pattern recognition receptors on immune cells. Some DAMPs are actively induced by cells undergoing immunogenic cell death, such as calreticulin, and adenosine triphosphate (ATP), whereas others are induced passively, such as high-mobility group box 1 (HMBG1). Some like members of the tumor necrosis factor (TNF)-family like FAS ligand (FASL), TNF and TNF-related apoptosis inducing ligand (TRAIL) can induce tumor-cell apoptosis upon the formation of immune synapses. These DAMPs play a beneficial role in anti-cancer therapy by interacting with the immune system [12, 13]. Chronic inflammation is typically associated with ovarian cancers, with high levels reactive oxygen species, cytokines, growth factors and inflammatory mediators [14]. An important member of cytokines is the interleukin-1 (IL-1) superfamily which has critical functions in proper maintenance of the innate and adaptive immune system [15]. Various genomic studies have shown that single nucleotide polymorphisms in the IL-1 superfamily can lead to higher susceptibility for immunological pathologies and disease presentation [15].

Medicinal plants have been traditionally used to treat numerous human health conditions and offer a vast resource as drug leads or novel therapeutic agents [16]. Despite the extensive biodiversity of medicinal plants around the world including Southeast Asia, there is scant documentation on the usage of fresh medicinal plants. Rapid urbanization poses a real threat to their natural habitat. Further, there is inadequate research on their pharmacological activities and scientific basis for their medicinal use. *Leea indica* (Burm. f.) Merrill, which belongs to the genus *Leea* and family Vitaceae, can be found in tropical and subtropical forests of Southeast Asia, China, India, and north Australia [17, 18]. In Singapore, the plant is distributed in the coastal areas, mangroves, secondary forests and the undergrowth of primary forests [19]. *L. indica* is also known as Bandicoot berry in English, or Yan Tuo 岩陀 in Chinese, Memali in Malay [17, 18]. The leaves, roots and fruits of *L. indica* have been traditionally used to treat a wide variety of ailments including cardiovascular diseases, cancer, diabetes, diarrhea, dysentery, eczema, fever, headache, and pain [18, 20, 21]. In vitro studies showed that the leaves of *L. indica* have various biological activities, including antihyperglycemic [22], antimicrobial [23], antioxidant [23], anticancer [24], anxiolytic [25], thrombolytic [26] and phosphodiesterase inhibitory effects [27]. Essential oils from the flowers may have antimicrobial activity [28], and the entire plant may have antioxidant and nitric oxide inhibitory activities [29]. In view that medicinal plants are good sources of novel therapeutics while treatment options for refractory ovarian cancer are limited and NK cell therapy looks promising, we wish to explore the effects of NK cell killing of ovarian cancer cells triggered by a phytoconstituent identified in a medicinal plant. We have previously shown that the maceration methanolic leaf extract of *L. indica* had good anti-proliferative activity against various human cancer cells, including ovarian cancer cells [30]. However, the effect of *L. indica* or its phytoconstituent on ovarian cancer cells and with chemotherapeutic drug oxaliplatin or NK cells are not known. Hence the objective of this study is to investigate the effects of *L. indica* leaves and its selected phytoconstituents on human ovarian cancer cells and in combination with oxaliplatin and NK cells.

## Methods and materials

### Plant source and preparation of leaf extracts

Fresh, healthy and mature leaves of *L. indica* leaves were obtained from the National University of Singapore Medicinal Plant Garden. A voucher specimen of *L. indica* (LI-0109) was deposited at the Department of Pharmacy Herbarium,  National University of Singapore. The plant name was checked with The World Flora Online http://www.worldfloraonline.org [31] and identified with reference to the “World Checklist of Selected Plant Families” and the journal article “*Leea* L. (Vitaceae) Of Singapore” [19]. The leaves were washed, air dried and blended using a dry grinder, and macerated using 70% *v/v* methanol [32]. The extracts were dried under vacuo and stored at 25 °C. All procedures were conducted in accordance to the guidelines:—https://www.biomedcentral.com/getpublished/editorial-policies#research+involving+plants.

### Isolation of chemical constituents from *L. indica* leaf extracts and chemical analyses

Leaf extracts from *L. indica* were prepared as described previously [32]. Briefly, the dried maceration 70% methanol crude leaf extract was dissolved in water and partitioned with *n*-hexane, dichloromethane and ethyl acetate to yield hexane, dichloromethane, ethyl acetate, water-soluble and water-insoluble fractions. These fractions were analyzed for their effects on cell viability and NK cell cytotoxicity. The crude extract and ethyl acetate fraction were also investigated for their effects on cytokine production. The ethyl acetate fraction was subjected to two column chromatographic separations in silica gel 60 using hexane, dichloromethane and methanol, followed by a final column chromatographic separation in silica gel 60 using hexane and ethyl acetate. At each step of the purification, concurrent WST-1 and NK cell cytotoxicity assays were performed. A fraction that displayed significant sensitization of OVCAR-5 ovarian cancer cells to NK cell-mediated killing was selected for further purification. Methyl gallate was isolated at a final gradient of 30% *v/v* ethyl acetate in hexane. To isolate gallic acid, the ethyl acetate fraction was subjected to a column chromatographic separation in silica gel 60 using hexane, dichloromethane and methanol, followed by a final column chromatographic separation in Sephadex LH-20 using water and methanol. Gallic acid was isolated at a final gradient of 20% *v/v* methanol in water. Commercial chemical standards of gallic acid and methyl gallate were purchased from Sigma-Aldrich (USA). Chemical analyses of the leaf extracts, fractions and isolated compounds were performed as described previously [32].

### General cell culture

Human advanced ovarian cancer cell lines OVCAR-5 (NCI Frederick, USA) and SK-OV-3 (ATCC, HTB-77), human monocytic cell line U937 (ATCC, CRL-1593.2), human NK cell line NK-92 (ATCC, CRL-2407) were purchased. Genetically modified K562-mb15-41BBL cell line was generated by Professor Dario Campana. OVCAR-5, SK-OV-3, U937 and K562-mb15-41BBL cells were grown in RPMI-1640 medium (ThermoScientific, USA) supplemented with 10% *v/v* heat-inactivated fetal bovine serum (FBS) (ThermoScientific, USA), while NK-92 cells were grown in RPMI-1640 medium containing 100 ng/mL rhIL-2 (Gibco, USA), 12.5% *v/v* FBS and 12.5% *v/v* horse serum (Gibco, Cat. no. 26050–088). All the cells were maintained at 37 °C and 5% CO_2_ in a humidified atmosphere. Methyl gallate and gallic acid were purchased from Sigma-Aldrich (USA), while clinical grade oxaliplatin was purchased (Eloxatin®, Sanofi, France).

For macrophage differentiation, U937 cells grown in in RPMI-1640 medium supplemented with 2% *v/v* FBS were treated with 5 ng/mL phobol-12-myristate-13-acetate (PMA) (Sigma-Aldrich, USA) for 24 h and washed with PBS as previously described [33]. These PMA-differentiated U937 macrophages were also referred to as U937 macrophages in this study.

The leaf extracts and chemical standards were dissolved in dimethyl sulfoxide (DMSO) (Sigma-Aldrich, USA) and diluted to the desired concentration before addition to cells. To examine the effect of leaf extract, fraction or standard compound on cytokine production, U937 macrophages were incubated for 6 h with the appropriate agent, and then activated overnight with 50 ng/mL lipopolysaccharide (LPS) (Sigma-Aldrich, USA) as previously mentioned [33].

Peripheral blood samples were obtained from discarded anonymized by-products of platelet donations from healthy adult donors at the Health Sciences Authority Blood Bank, Singapore. Studies were performed with approval from the Institutional Review Board, National University of Singapore. Human NK cells were expanded and activated according to the patented methods US 7,435,596 B2 and US 8,026,097 B2 that were established by Professor Dario Campana [34, 35]. Briefly, mononuclear cells collected by centrifugation on a Ficoll-Paque Plus (GE Healthcare Life Sciences) were washed twice in RPMI-1640 medium. To expand CD56 + CD3- NK cells, peripheral blood mononuclear cells and genetically modified K562-mb15-41BBL cell line (E:T = 1:1) were cultured with CellGro SCGM medium (CellGenix, Germany) supplemented with 10% *v/v* FBS and 10 IU/mL human IL-2 (Affymetrix eBioscience, USA). Fresh medium was topped up every two days with IL-2. After 9 days of co-culture, CD3 + T cells were depleted by human CD3 MicroBeads (Miltenyi Biotec, Germany) from autoMACS Separator (Miltenyi Biotec, Germany), generating CD56 + CD3- NK cells with more than 95% purity.

### Determination of cell viability by water soluble tetrazolium salts (WST-1) assay

This assay was performed as described previously [33, 36]. Briefly, exponentially growing cells were plated in 96-well plates at 3 × 10^4^ cells/100 µL (OVCAR-5, U937), or 7 × 10^3^ cells/100 µL (SK-OV-3). U937 cells were treated with PMA for 24 h to differentiate into macrophages and washed with PBS. These differentiated U937 macrophage cells and the adherent ovarian cancer cells were treated with the appropriate agent (extract/drug/vehicle control) for 48 h, and untreated cells were used as controls. After 48 h, the media was aspirated and replaced with 10% *v/v* WST-1 (Roche, Switzerland) for 1 h. The formazan dye produced was quantified at 440 nm against a reference wavelength of 650 nm using a microplate reader (Tecan Infinite M200 PRO, Switzerland). Cell viability was expressed as a percentage of the control cells. The IC_50_ value (i.e. concentration of extract/compound required to inhibit 50% growth of cells) from cell viability assay was used as a parameter for anti-proliferative potency [37, 38], while the IC_20_ value (i.e. concentration of extract required to inhibit 20% growth of cells) was taken as an indicator for non-toxic dose of test sample [37]. The IC_50_ and IC_20_ values were determined using GraphPad Prism 9 (GraphPad Software, Inc., USA). The results were generated from three independent experiments and each experiment was performed in 5 replicates.

### Evaluation of cytokine production by ELISA assay

The production of TNF-α and IL-1β cytokines were measured in U937 macrophages by ELISA as previously described [33]. Briefly, U937 cells were plated in 6-well plates (Costar, USA) at 1 × 10^6^ cells per well, treated with PMA for 24 h to differentiate into macrophages and washed with PBS. These PMA-differentiated cells were incubated with the appropriate agents as described above. Cell supernatant was collected at the end of incubation and analysed for the level of cytokines IL-1β and TNF-α using ELISA kit from Quantikine (R&D Systems, Minneapolis, USA) according to manufacturer’s instructions. Briefly, standards and samples were added to wells pre-coated with antibodies for 2 h, washed, and incubated with cytokine conjugate for 1 h. After washing, substrate solution was added for 20 min, followed by stop solution. The cytokine level present was quantified at 450 nm against a reference wavelength of 540 nm using a microplate reader (Tecan Infinite M200 PRO, Switzerland) and absolute concentrations of cytokines were interpolated from their respective standard curves. Standard curves were achieved using standard concentrations of the human IL-1β and TNF-α based on manufacturer’s instructions. The results were generated from three independent experiments.

### NK cell cytotoxic activity assay

This assay was performed as described previously [36]. Ten million cancer cells were washed several times after compound pre-treatment to deplete any minimum residue compound. Cells were labelled with a red fluorescent dye PKH-26 (Sigma-Aldrich, USA) that bound irreversibly to the cell membrane. After incubation for 15–30 min at room temperature, labelled cells were washed three times with RPMI-1640 medium and the viability of target cells was evaluated by trypan blue exclusion counting. Ten thousand viable OVCAR-5 and SK-OV-3 target cells were attached to a 96-well, flat-bottomed plate which was pre-coated with poly-L-lysine (Sigma-Aldrich, USA). Target cells were co-cultured with activated NK cells for 8 or 12 h, respectively, at the indicated effector-to-target (E:T) ratios. For NK-92 cells, the co-culture duration was 12 h. After incubation, lysed cells were gently washed away with PBS. Multiple reads per well were obtained with fluorescence at excitation wavelength 540 nm and emission wavelength 590 nm using a multimode microplate reader (Tecan Spark®, Switzerland). Cytotoxicity was assessed by measuring the viability of PKH-26 positive target cells that were still attached on the plate. The percentage of viability for target cells was calculated as (target plus effector cells fluorescence – maximum lysis fluorescence) / (target cells alone fluorescence – maximum lysis fluorescence) × 100%. The percentage of specific lysis for NK cells was calculated as 100% – % viability [36]. Experiments were performed in triplicate and at least three independent experiments were done. To investigate the tumor cell proliferation dynamics of ovarian cancer cells in the presence of NK-92 cells, cancer cells were co-cultured with NK-92 at E:T ratio of 1:4, media was replaced daily partially and cell viability was evaluated by trypan blue exclusion counting every 3 days.

### Antibody staining and flow cytometry

Anti-human phycoerythrin (PE)-conjugated antibodies used in this study were: anti-CD112 (TX31, IgG1, BioLegend), anti-CD155 (TX24, IgG2a, BioLegend), anti-MIC-A/B (6D4, IgG2a, BioLegend), anti-ULBP-1 (Clone 170818, IgG2a, R&D Systems), anti-ULBP-2 (Clone 165903, IgG2a, R&D Systems), anti-ULBP-3 (Clone 166510, IgG2a, R&D Systems), anti-DR4 (CD261, TRAIL-R1, Clone DJR1, BioLegend), anti-DR5 (CD262, TRAIL-R2, Clone DJR2-4, BioLegend). The assay was performed as described previously [36]. Briefly, after drug treatment, cancer cells were incubated with antibodies in fluorescence-activated cell sorting (FACS) solution on ice for 30 min. Cells were washed three times with FACS solution and then fixed with 2% paraformaldehyde (Sigma-Aldrich, USA). For intracellular staining, cells were first permeabilized and fixed in Cytofix/Cytoperm™ solution (BD Pharmingen) based on manufacturer’s instructions, washed and then incubated with antibody to intracellular targets for 30 min on ice. After staining, cell samples were acquired and recorded on a FACSCalibur (BD Biosciences, USA), and data was analyzed with BD CellQuest Pro and FlowJo software (Tree Star). For investigating the expression levels of stress ligands by flow cytometry, samples were stained and acquired together on the flow cytometer on the same day, using the same voltage settings. Data were presented as relative mean fluorescence intensity (MFI) calculated (MFI of treated cancer cells) / (MFI of non-treated cancer cells), and control cells (i.e. non-treated cancer cells) were taken as relative MFI of 1.

### Statistical analyses

All statistical analyses were performed with GraphPad Prism 9 (San Diego, California, USA). The correlation of ovarian cancer cell phenotypes expressing stress ligands and death receptors were analyzed with Student’s *t*-test. For evaluating combination therapy of methyl gallate and NK cells on the proliferation of ovarian cancer cells, one-way ANOVA with Bonferroni’s multiple comparison test was applied. For analyses of NK cell cytotoxicity, multiple comparisons were performed using two-way ANOVA with a Bonferroni test. For assessing the fold change of TNF-α and IL-1β levels, one-way ANOVA was used. Mean and SD of data from triplicate experiments were applied. Error bars show standard deviation (SD) as indicated in legend, and *p* < 0.05 was considered statistically significant. * indicates *p* < 0.05, ** *p* < 0.01, *** *p* < 0.001, ns denotes not significant.

An overall flow chart of the methods is shown in Supplementary Fig. 1.

## Results

### *L. indica* leaf extract promoted sensitivity of ovarian carcinoma cells lines to NK cell-mediated cytolysis

**Fig. 1:**
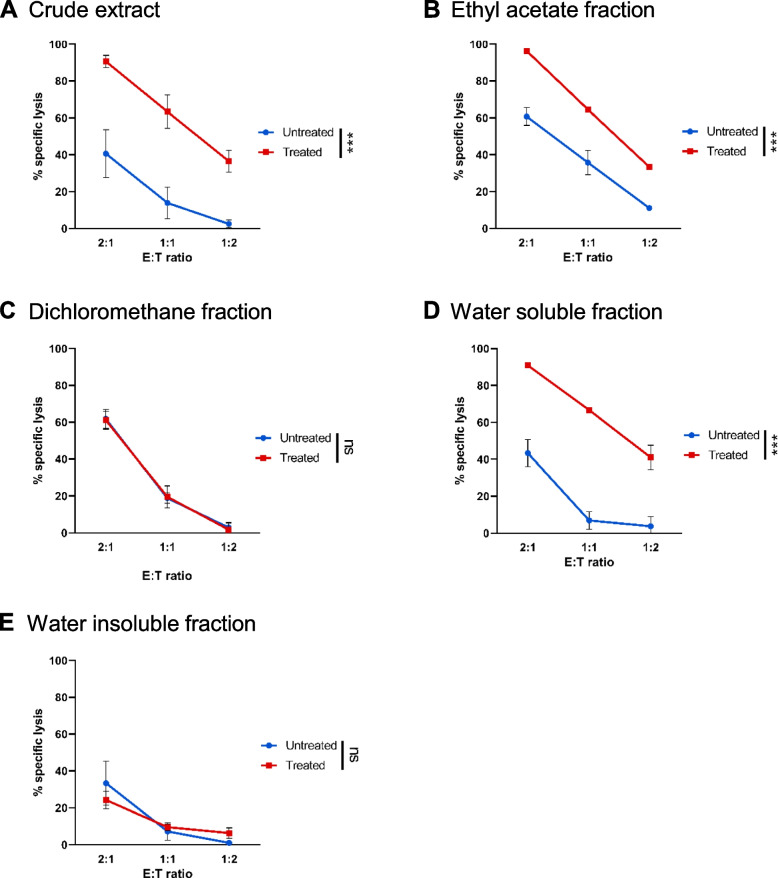
*L. indica* leaf extract pretreated ovarian cancer cells showed enhanced sensitivity to cytolysis by activated NK cells. OVCAR-5 cells were pretreated overnight with or without 0.3 mg/mL (**A**) crude extract, (**B**) ethyl acetate fraction, (**C**) dichloromethane fraction, (**D**) water soluble fraction and (**E**) water insoluble fraction of *L. indica*. Subsequently cells were co-cultured with activated NK cells at the various E:T ratios. Cytotoxicity assay was determined by measuring the viability of PKH-26 labelled target cancer cells on 96-well plate. Experiment was performed in triplicates. Results from one representative experiment of three are shown and presented as mean ± SD. Blue line represents untreated cells, while red line represents treated cells. ***p < 0.001; ns, not significant

Crude methanolic extract of *L. indica* leaves was partitioned by liquid–liquid partitioning to yield hexane, dichloromethane, ethyl acetate and water-soluble fractions as well as water-insoluble fraction. Human ovarian cancer OVCAR-5 cells were treated with various concentrations of crude leaf extract and fractions for 48 h and cell viability was measured by WST-1 assay. Their IC_50_ values are shown in Table 1. Amongst the fractions, the ethyl acetate fraction showed the lowest IC_50_ value (IC_50_ = 59.7 ± 1.9 µg/mL), followed by water-soluble fraction (IC_50_ = 260.8 ± 17.9 µg/mL). The IC_50_ value of the ethyl acetate fraction was about half of that of the crude extract (IC_50_ = 122.5 ± 13.8 µg/mL). Due to its low yield, the hexane fraction was not further studied. The crude leaf extract and four fractions were next assessed for their effects on human ovarian cancer cells in NK cell-mediated cytotoxicity with activated NK cells. Ovarian cancer cells were washed three to five times to deplete minimum residue of each test sample after treatment with either the extract or fraction. Compared to untreated cells, we found pretreatment of ovarian cancer OVCAR-5 cells with crude leaf extract significantly increased the susceptibility of the cancer cells to cytolysis by activated NK cells (*p* < 0.001, Fig. 1A). Similar phenomenon was observed for the ethyl acetate and water-soluble fractions. OVCAR-5 cells that were pretreated with either ethyl acetate fraction (*p* < 0.001, Fig. 1B) or water-soluble fraction (*p* < 0.001, Fig. 1D) exhibited enhanced susceptibility to cytotoxicity with activated NK cells, compared to untreated group. There was no significant difference between untreated OVCAR-5 cells and cells pretreated with dichloromethane fraction (Fig. 1C) or water insoluble fraction (Fig. 1E). Taken together, ovarian cancer cells pretreated with ethyl acetate fraction showed enhanced susceptibility to activated NK cell-mediated cytolysis, and the ethyl acetate fraction showed the strongest anti-proliferative activity in ovarian cancer cells relative to the other fractions. Therefore, the ethyl acetate fraction was subjected to subsequent further rounds of column chromatography purification, accompanied by concurrent WST-1 and NK cell cytotoxicity assays. At each step, a fraction that significantly sensitized the ovarian cancer cells to NK cell-mediated killing was selected for further purification. This sequential process of bioassay-guided fractionation led to the isolation and identification of gallic acid and methyl gallate [32].

**Table 1:** IC_50_ values of crude leaf extract and fractions of *L. indica* in OVCAR-5 cells

LI extract and fractions	IC_50_ values (µg/mL)
Crude extract	122.5 ± 13.8
Dichloromethane fraction	1542.0 ± 115.3
Ethyl acetate fraction	59.7 ± 1.9
Hexane fraction	818.6 ± 74.8
Water insoluble fraction	479.9 ± 52.7
Water soluble fraction	260.8 ± 17.9

Data are presented as mean ± SD from 3 independent experiments, each carried out in triplicates

### Methyl gallate but not gallic acid enhanced sensitivity of ovarian carcinoma to NK cell-mediated cytolysis

**Fig. 2:**
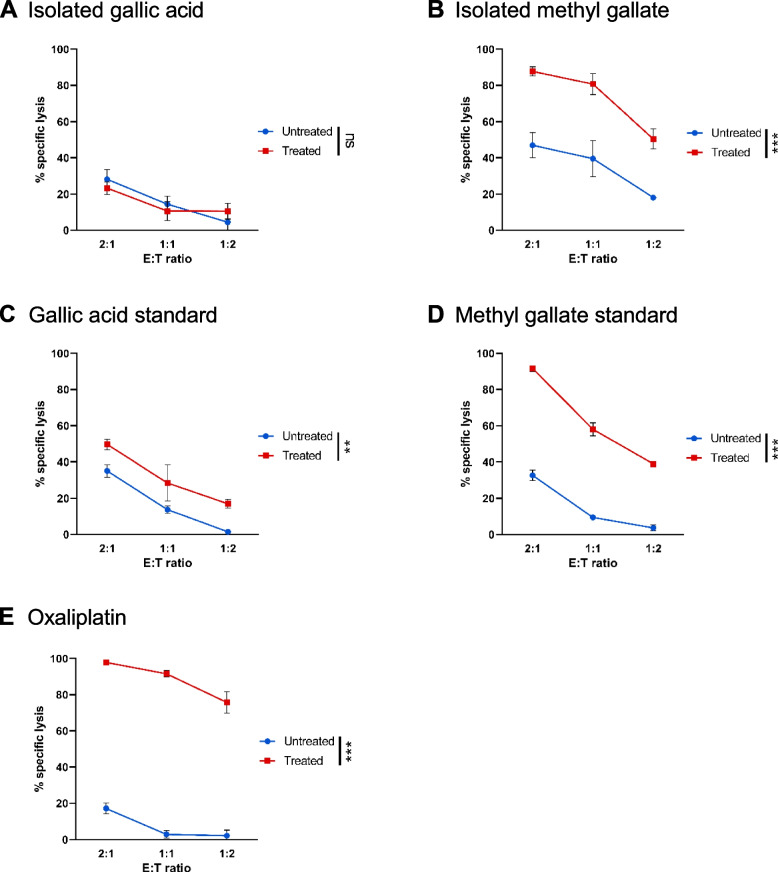
Methyl gallate pre-treatment increased susceptibility of ovarian cancer cells to NK cell-mediated cytolysis. OVCAR-5 cells were pretreated overnight with or without (**A**) 0.03 mg/mL isolated gallic acid, (**B**) 0.1 mg/mL isolated methyl gallate, (**C**) 0.03 mg/mL gallic acid standard, (**D**) 0.1 mg/mL methyl gallate standard, (**E**) 20 µM oxaliplatin, and then co-cultured with activated NK cells at the various E:T ratios. Cytotoxicity assay was determined by measuring the viability of PKH-26 labelled target cancer cells on 96-well plate. Experiment was performed in triplicates. Results from one representative experiment of three are shown and presented as mean ± SD. Blue line represents non-treated cells, while red line represents treated cells. **p < 0.01; ***p < 0.001; ns, not significant

Gallic acid and methyl gallate were analyzed for their anti-proliferative effects in OVCAR-5 ovarian cancer cells. The IC_50_ values of gallic acid and methyl gallate in OVCAR-5 cells were 14.4 ± 0.6 µg/mL (84.9 ± 3.4 µM) and 93.7 ± 3.3 µg/mL (509.0 ± 17.7 µM), respectively. The effects of gallic acid and methyl gallate on human ovarian cancer cells followed by co-culture with activated NK cells were next determined. We chose a concentration as close to the IC_50_ value as possible, namely 0.03 mg/mL gallic acid and 0.1 mg/mL methyl gallate. We examined both the isolated compounds and their respective chemical standards, and the results are shown in Fig. 2. Oxaliplatin, a third-generation platinum drug which is known to augment sensitivity of colon cancer [39] and ovarian carcinoma [36] to NK cell-mediated cytolysis, was used as positive control (*p* < 0.001, Fig. 2E). Compared to untreated cells, we found no significant difference in cytolysis of OVCAR-5 cells that were pretreated with the isolated gallic acid (Fig. 2A). In ovarian cancer cells pretreated with the chemical standard gallic acid, there was low level of NK cell cytolysis (*p* < 0.001, Fig. 2C). In contrast, the isolated methyl gallate (*p* < 0.01, Fig. 2B) and its chemical standard (*p* < 0.001, Fig. 2D) significantly elevated the susceptibility of ovarian cancer OVCAR-5 cells to cytolysis by expanded NK cells. These results suggest methyl gallate significantly enhanced the susceptibility of ovarian cancer cells to specific NK-cell mediated cytolysis, whereas gallic acid had low to no effect at the concentration investigated.

### *L. indica* extract and methyl gallate upregulated the expression of stress ligands for NK cell receptors in ovarian cancer cells

**Fig. 3:**
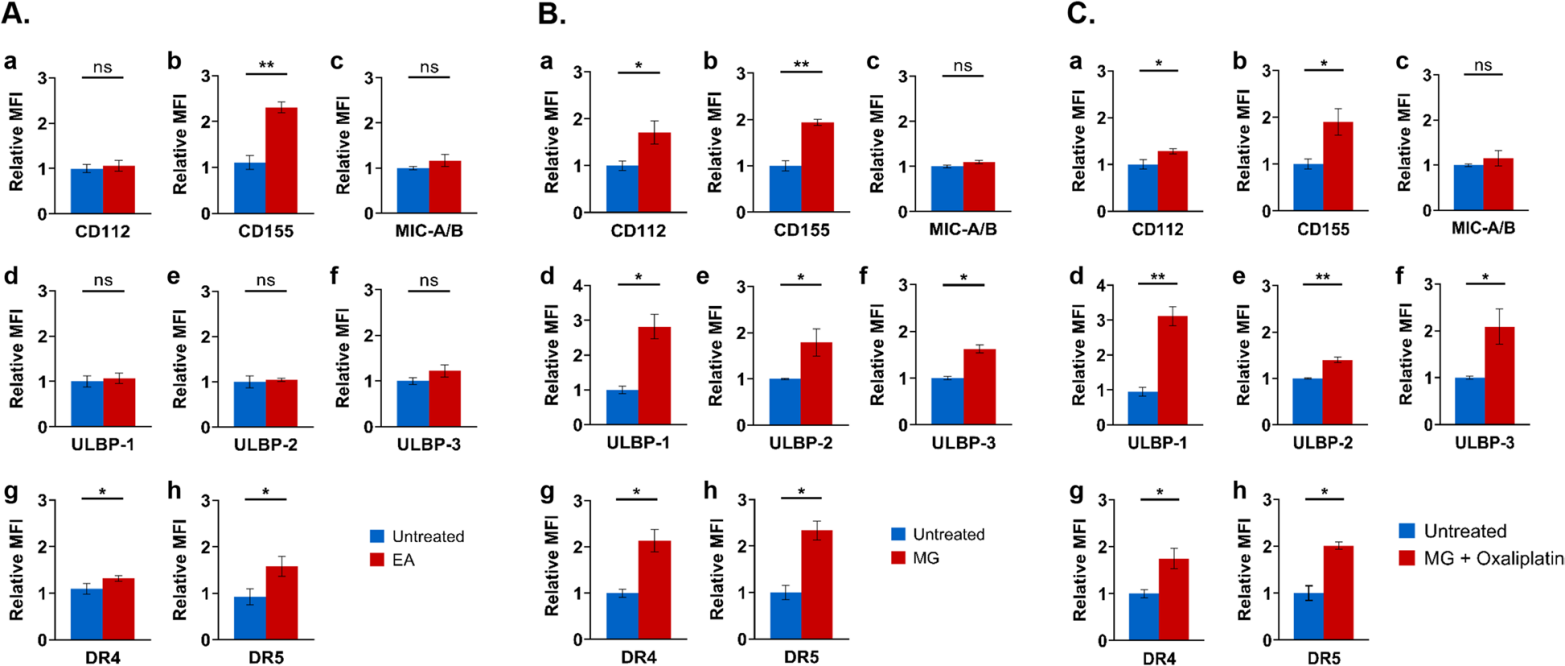
Increased expression of stress ligands for NK cell receptors on ovarian cancer cells after treatment with (**A**) ethyl acetate fraction of *L. indica*, (**B**) methyl gallate, and (**C**) combination of methyl gallate and oxaliplatin. OVCAR-5 cells were treated for 48 h with or without (**A**) *L. indica* ethyl acetate fraction (EA, 0.3 mg/mL), (**B**) methyl gallate (MG, 0.1 mg/mL), or (**C**) combination of methyl gallate (MG, 0.1 mg/mL) and oxaliplatin (10 µM), and then phenotype analyzed by FACS for the indicated ligands of NK cells: (a) CD112, (b) CD115, (c) MIC-A/B, (d) ULBP-1, (e) ULBP-2, (f) ULBP-3, (g) DR4 (TRAIL-R1), and (h) DR5 (TRAIL-R2). The relative mean fluorescence intensities of each stress ligand were compared between untreated cells (blue bars) and treated cells (red bars), and results presented are mean ± SD of three independent experiments. *p < 0.05; **p < 0.01; ns, not significant

To examine in ovarian cancer OVCAR-5 cells the ability of *L. indica* extract-mediated induction of activating ligands for NK cell receptors (DNAM-1 and NKG2D), the cancer cells were treated with the ethyl acetate fraction, and subsequently analyzed for the stress ligands by immunofluorescence conjugated specific monoclonal antibodies. We found that the ethyl acetate fraction of *L. indica* significantly induced higher expression of CD155 (*p* < 0.01), DR4 (TRAIL-1, *p* < 0.05) and DR5 (TRAIL-2, *p* < 0.05) in cancer cells treated with ethyl acetate fraction compared to untreated cells (Fig. 3A, Supplementary Fig. 2A). The other ligands for NK cell receptors did not show any significant difference between treated and untreated cancer cells (Fig. 3A, Supplementary Fig. 2A). We next investigated the ability of methyl gallate to mediate the induction of activating ligands for NK cell receptors DNAM-1 and NKG2D in ovarian cancer cells. Compared to untreated cells, methyl gallate-treated cells displayed significant induction of CD112, CD155, ULBP-1, ULBP-2, ULBP-3, DR4 (TRAIL-1), and DR5 (TRAIL-R2) (*p* < 0.05, *p* < 0.01, Fig. 3B, Supplementary Fig. 2B). Both DR4 and DR5 are death receptors for tumor necrosis factor-related apoptosis-inducing ligands (TRAIL), a cytokine produced by NK and T cells that exhibit specific tumoricidal activity against a variety of tumors. We had previously shown that oxaliplatin at 20 µM upregulated the expression of stress ligands for NK cell receptors in OVCAR-5 cells and enhanced NK cell cytolysis of ovarian cancer OVCAR-5 cells [36]. Oxaliplatin, like all other chemotherapeutic agents when used at high doses, are known to have clinically adverse side effects such as peripheral neuropathy and nausea [40–42]. We therefore chose a lower concentration of oxaliplatin (i.e. 10 µM), and asked if a combination of methyl gallate with low dose oxaliplatin had any effect on the stress ligand expression on ovarian cancer cells. Figure 3C (Supplementary Fig. 2C) shows that cancer cells treated with combination of methyl gallate and low dose oxaliplatin (i.e. 10 µM) displayed an overall significant induction of CD112, CD155, ULBP-1, ULBP-2, ULBP-3, DR4, and DR5 (*p* < 0.05, *p* < 0.01). We also investigated the effect of gallic acid treatment on the levels of stress ligands in ovarian cancer cells. We found that gallic acid-treated OVCAR-5 cells did not show any significant difference in the expression of these stress ligands (Supplementary Fig. 3). Taken together, these data (Figs. 2 and 3, Supplementary Figs. 2 and 3) indicate that ovarian cancer cells treated with methyl gallate, or with combination of methyl gallate and low concentration of oxaliplatin (i.e. 10 µM), showed increased expression of stress ligands for NK cell receptors and concomitantly enhanced sensitivity to NK cell-mediated cytolysis. Conversely, ovarian cancer cells treated with gallic acid showed no significant difference in the expression of these stress ligands and marginal susceptibility to NK cell killing.

### Combination treatment of methyl gallate and oxaliplatin augmented susceptibility of ovarian tumor cells to NK cell-mediated cytolysis

**Fig. 4:**
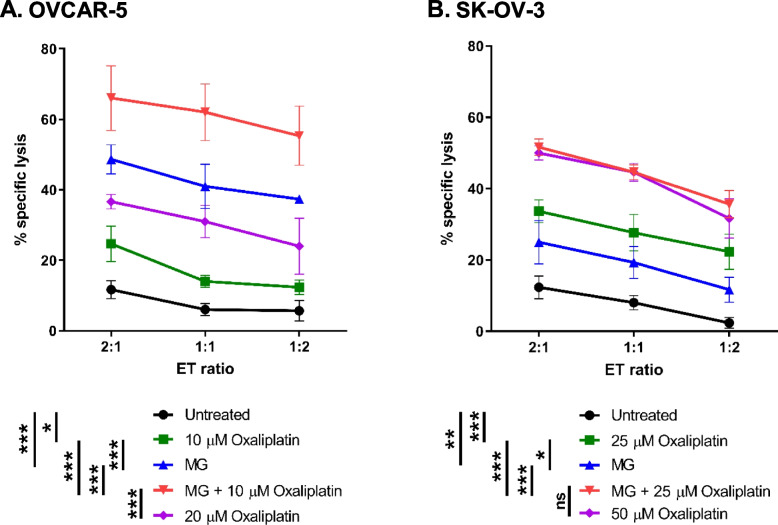
Combination treatment of methyl gallate and oxaliplatin augmented susceptibility of ovarian cancer cells to NK cell-mediated cytolysis. **A** OVCAR-5 cells were pretreated for 24 h with or without 20 μM oxaliplatin, 10 μM oxaliplatin, 0.1 mg/mL methyl gallate (MG) alone, or combination of 0.1 mg/mL MG and 10 μM oxaliplatin. **B** SK-OV-3 cells were pretreated for 48 h with or without 50 μM oxaliplatin, 25 μM oxaliplatin, 0.02 mg/mL methyl gallate (MG) alone, or combination of 0.02 mg/mL MG and 25 μM oxaliplatin. Cells were then co-cultured with NK-92 cells at the various E:T ratios. Experiment was performed in triplicates. Results from one representative experiment of three are shown and presented as mean ± SD. *p < 0.05; **p < 0.01; ***p < 0.001; ns, not significant

Methyl gallate was analyzed for its anti-proliferative effects in SK-OV-3 cells, and the IC_50_ value of methyl gallate in SK-OV-3 cells was found to be 21.7 ± 2.4 µg/mL (117.5 ± 12.8 µM). Clearly, the IC_50_ value of methyl gallate in SK-OV-3 cells was lower than that of the IC_50_ value in OVCAR-5 cells (93.7 ± 3.3 µg/mL or 509.0 ± 17.7 µM). We chose a concentration of methyl gallate as close to the IC_50_ value as possible, namely 20 µg/mL and 100 µg/mL for SK-OV-3 and OVCAR-5 cells, respectively. We had previously shown that oxaliplatin at 20 µM and 50 µM significantly increased specific cytolysis of activated NK cells against ovarian cancer OVCAR-5 and SK-OV-3 cells respectively [36]. As high drug concentration is typically associated with clinically undesirable side effects such as acute neuropathy [41, 42], we studied oxaliplatin at low concentration of 10 µM and 25 µM for ovarian cancer OVCAR-5 and SK-OV-3 cells respectively. We investigated the effects of methyl gallate combined with oxaliplatin or methyl gallate alone in these ovarian cancer cells on their sensitivity to NK cell-mediated cytotoxicity. Compared to untreated cells, we found OVCAR-5 and SK-OV-3 cancer cells pretreated with 10 µM (*p* < 0.05, Fig. 4A) and 25 µM (*p* < 0.001, Fig. 4B) oxaliplatin respectively exhibited increased susceptibility to NK-92 cells. Compared to untreated cells, pre-treatment of cancer cells with methyl gallate showed the OVCAR-5 (*p* < 0.001, Fig. 4A) and SK-OV-3 cancer cells (*p* < 0.01, Fig. 4B) were relatively sensitive to NK cell-mediated cytolysis, albeit at low levels. Interestingly, pre-treatment of OVCAR-5 cancer cells with combined methyl gallate and 10 µM oxaliplatin greatly augmented the susceptibility of these cancer cells to NK cell cytolysis (*p* < 0.001, Fig. 4A). Similarly, pre-treatment of SK-OV-3 cancer cells with combined methyl gallate and 25 µM oxaliplatin greatly enhanced the susceptibility of the cancer cells to NK cell cytolysis (*p* < 0.001, Fig. 4B). These results suggest that treatment of ovarian cancer cells with combined methyl gallate and low concentration of oxaliplatin can enhance the susceptibility of ovarian cancer cells to NK cell-mediated cytolysis.

### Pre-treatment with methyl gallate reduced ovarian cancer cell proliferation when co-cultured with NK cells

**Fig. 5:**
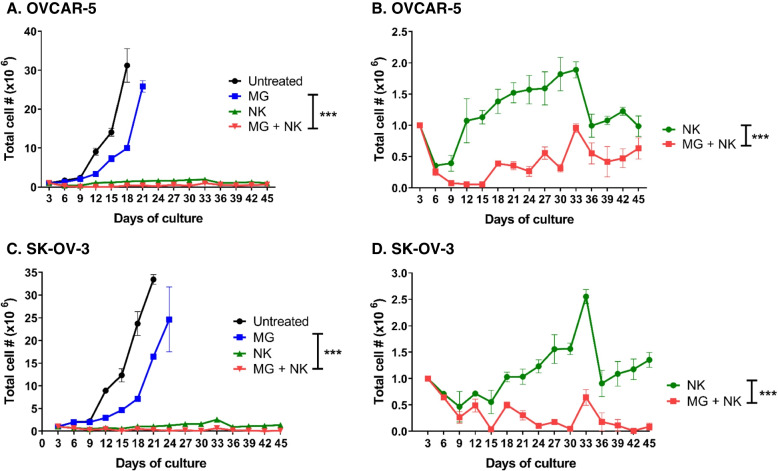
Combination therapy of methyl gallate and NK-92 cells suppressed re-proliferation of ovarian cancer cells. **A**, **B** OVCAR-5 cells were pretreated for 24 h with 0.1 mg/mL methyl gallate (MG), and (**C**, **D**) SK-OV-3 cells were pre-treated for 48 h with 0.02 mg/mL methyl gallate (MG). Cells were then co-cultured in the presence or absence of NK-92 cells at E:T ratio of 1:4. Untreated cells co-cultured in the presence or absence of NK-92 cells served as control. Cells were counted every 3 days. Values presented are the average ± SD of three independent experiments performed in triplicates. ***p < 0.001

To measure tumor growth dynamics of the ovarian cancer cells in the presence of NK-92 cells, we chose a very low E:T ratio of 1:4 and monitored the proliferation of these cancer cells which had been pre-treated with methyl gallate. Untreated cancer cells or cancer cells pre-treated with methyl gallate were grown in the absence of NK-92 cells and served as control groups. Results showed that in the absence of any treatment, ovarian cancer cells grew exponentially as expected (Fig. 5A and C). OVCAR-5 cancer cells that were pre-treated with methyl gallate, and then subsequently cultured in the absence of NK-92 cells, recovered their propensity to grow by day 15 (Fig. 5A). Similarly, SK-OV-3 cancer cells that were pre-treated with methyl gallate, and then subsequently cultured in the absence of NK-92 cells, showed signs of exponential growth by day 18 (Fig. 5C). In contrast, ovarian cancer cells that received treatment with both methyl gallate and NK-92 cells in a sequential manner were unable to initiate their growth potential (*p* < 0.001, Fig. 5B and D). Without methyl gallate treatment, OVCAR-5 and SK-OV-3 cancer cells that were co-cultured with low E:T ratio of NK-92 cells gradually showed signs of tumor cell proliferation, although at much lower levels compared with untreated tumor cells that were grown in the absence of NK cells (Fig. 5B and D). A schematic figure of some key findings is presented in Supplementary Fig. 4.

### *L. indica* extract and methyl gallate suppressed TNF-α and IL1-β cytokine release

**Fig. 6:**
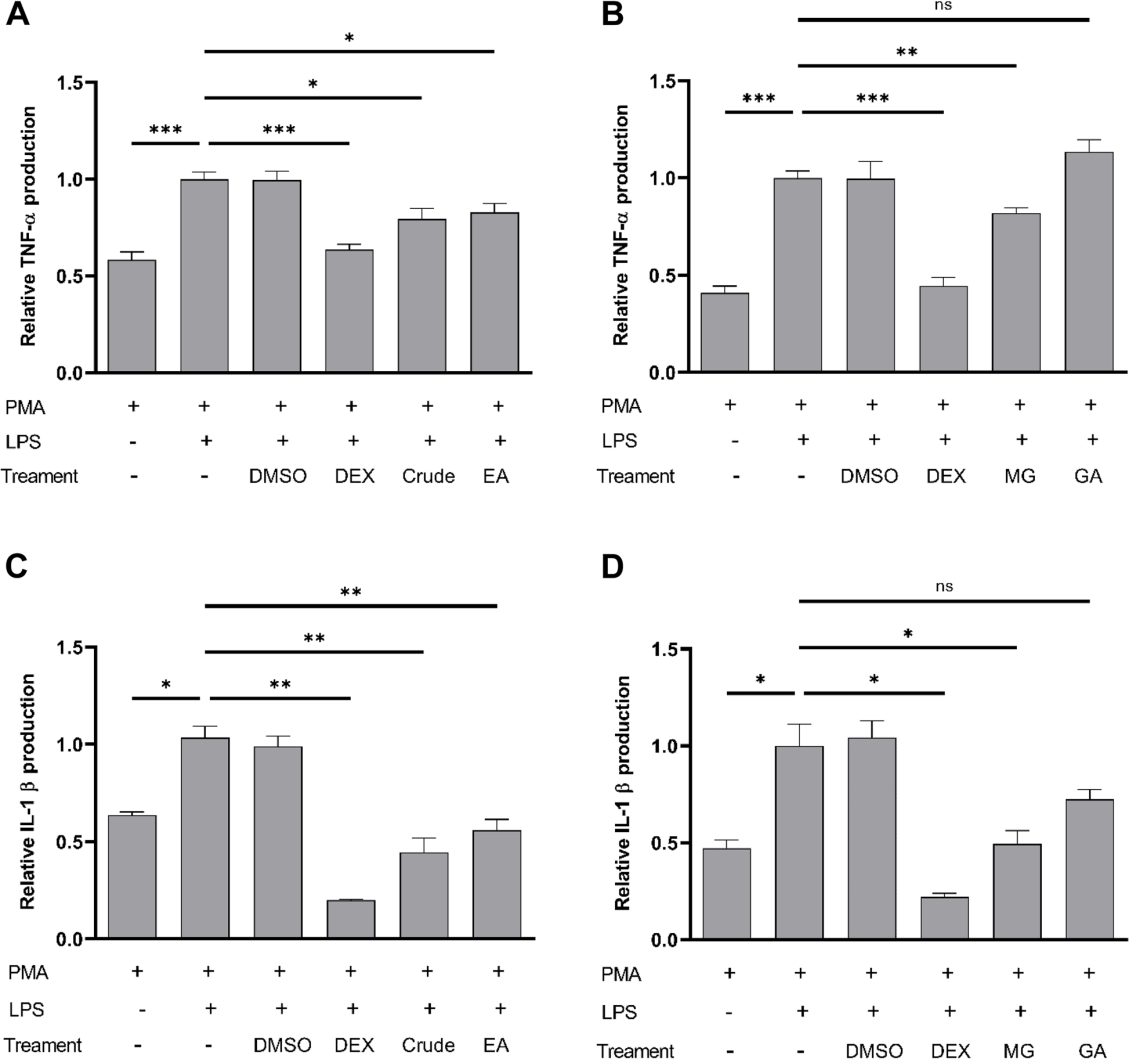
*L. indica* crude leaf extract, ethyl acetate fraction, and methyl gallate suppressed TNF-α and IL-1β production by human U937 macrophages. Fold change of TNF-α (**A**, **B**) and IL-1β (**C**, **D**) production relative to control in the supernatant of human U937 macrophages measured by ELISA. Cells were pretreated for 5 h with or without 40 μg/mL *L. indica* crude leaf extract, 10 μg/mL *L. indica* ethyl acetate fraction (EA), 38 μM methyl gallate (MG) or 38 μM gallic acid (GA), followed by LPS stimulation. Cytokine production by cells treated with both PMA and LPS was taken as 1. Dexamethasone (DEX) at 64.4 ng/mL was used as positive control, while DMSO was used as negative control. Results are presented as mean fold change ± SD of three independent experiments. *p < 0.05; **p < 0.01; ***p < 0.001; ns, not significant

We investigated the crude leaf extract and the ethyl acetate fraction of *L. indica* as well as methyl gallate and gallic acid in PMA-differentiated U937 cells for their effects on cytokine release. We first evaluated the crude leaf extract, ethyl acetate fraction, methyl gallate and gallic acid in U937 macrophages for their potential cytotoxicity using WST-1 cell viability assay. We found that *L. indica* crude leaf extract did not result in any appreciable difference in the cell viability of U937 macrophages even up to 100 µg/mL, while the ethyl acetate fraction displayed an IC_50_ value of 20.17 ± 0.46 µg/mL, with IC_20_ value of 9.7 ± 1.3 µg/mL. Methyl gallate and gallic acid displayed IC_50_ values of 8.2 ± 0.6 µg/mL (45.0 ± 3.3 µM) and 23.8 ± 2.5 µg/mL (139.8 ± 14.8 µM) in U937 macrophages respectively. The IC_20_ values of methyl gallate and gallic acid were 5.4 ± 0.4 µg/mL (29.3 ± 2.4 µM) and 18.0 ± 1.8 µg/mL (105.6 ± 10.9 µM) respectively. A criterion for examining the inflammatory effects of the leaf extract was that the concentration of leaf extract used should be the largest one in which the cells remained viable. We chose to use the IC_20_ values of the extracts [37], which represented the concentration at which at least 80% of the cell population was alive. Based on their different IC_20_ values, we studied the ethyl acetate fraction at 10 µg/ml, and methyl gallate and gallic acid at 38 µM. In the absence of any treatment, U937 cells produced very low levels of TNF-α and IL-1β. Treatment with PMA significantly increased the production of TNF-α and IL-1β in U937 macrophages (Fig. 6). Stimulation of PMA-differentiated cells (also referred here as U937 macrophages) with LPS further doubled the production of both TNF-α (*p* < 0.001, Fig. 6A) and IL-1β (*p* < 0.05, Fig. 6C) compared to PMA treatment only. As expected, the increased TNF-α and IL-1β levels were abolished upon pre-incubation of cells with dexamethasone, a corticosteroid known to alleviate inflammatory conditions (Fig. 6). Interestingly, pre-incubation of U937 macrophages with either crude leaf extract or ethyl acetate fraction significantly suppressed TNF-α (*p* < 0.05, Fig. 6A) and IL-1β levels (*p* < 0.01, Fig. 6C). Pre-treatment of U937 macrophages with 38 µM methyl gallate significantly inhibited the production of TNF-α (*p* < 0.01, Fig. 6B) and IL-1β (*p* < 0.05, Fig. 6D). However, pre-treatment of U937 macrophages with 38 µM gallic acid did not show any significant difference on TNF-α and IL-1β levels (Fig. 6B and D).

## Discussion

*L. indica* is traditionally used to treat intestinal cancer and uterus cancer [43] and the leaf extracts of *L. indica* showed anticancer activity against Ehrlich Ascites Carcinoma (EAC) cells in Swiss albino mice, cervical epidermoid (Ca Ski) and most other human cancer cell lines [24, 25, 30]. Mollic acid arabinoside and mollic acid xyloside identified from the ethanol leaf extract were reported to be responsible for the cytotoxic effects against human cervical Ca Ski cancer cells [44], possibly via the stimulation of mitochondria-mediated apoptosis [45]. However, it is unclear if there are also other phytoconstituent(s) responsible and other mechanisms involved. Herein we report for the first time that leaf extracts of *L. indica* increased the susceptibility of human ovarian cancer cells to NK cell-mediated cytotoxicity (Fig. 1). The leaf extracts also suppressed the levels of TNF-α and IL-1β cytokines in human U937 macrophages (Fig. 6). We demonstrate for the first time that methyl gallate isolated and identified in *L. indica* can enhance the sensitivity of human ovarian cancer cells to NK cell-mediated cytotoxicity (Figs. 2 and 4), and this increased cytolysis was likely due to the associated elevated expression of various stress ligands for NK cell receptors (i.e. DNAM-1 and NKG2D) on these cancer cells (Fig. 3B, Supplementary Fig. 2B). Cancer cells pretreated with methyl gallate and subsequently cultured in the presence of NK cells were unable to initiate their growth potential (Fig. 5). Combined methyl gallate with low concentration of chemotherapeutic drug oxaliplatin enhanced the levels of stress ligand expression on tumor cells (Fig. 3C, Supplementary Fig. 2C), and this was accompanied by higher susceptibility to NK cell-mediated cytolysis (Fig. 4).

It is well documented that although NK cells are innate immune cells that play crucial roles in immunosurveillance and eliminating tumors, NK cell function is often impaired during tumor development and progression due to the presence of multiple immunosuppressive factors in the tumor microenvironment [46, 47]. Tumour variants can evade NK cell attack by mechanisms such as defective expression of activating ligands. Tumours may also upregulate ligands for inhibitory receptors and/or lose ligands for activating receptors, causing cells to be resistant against NK cell-mediated killing. For instance, high levels of circulating soluble MIC-A/B were associated with poor prognosis in a number of cancer types including colorectal, ovarian, liver, lung and prostate cancers [48, 49]. Compounds that enhance NK cell-mediated lysis of cancer cells are therefore highly beneficial and valuable, and they include bortezomib [50], doxorubicin [51] and oxaliplatin [52]. For example, the chemotherapeutic drug doxorubicin or the proteasome inhibitor bortezomib can trigger the upregulation of activating ligands for NKG2D receptor and DNAM-1 on multiple myeloma cells, thereby sensitizing them to NK cell-mediated lysis [53]. Bortezobmib inhibited proliferation of liver cancer cells and increased MIC-A/B expression (50). Natural products that are able to induce immunogenic cell death could represent novel lead compounds for cancer therapy. Some examples of reported natural products that induce immunogenic cell death include digoxin from *Digitalis* species and capsaicin from *Capsicum* species, as well as those derived from marine organisms such as *Spirulina maxima* [54], resveratrol [55], daphnetin [56], and stemphol [57]. Resveratrol, a naturally occurring plant polyphenol, sensitized human leukemia KG-1a cells to NK cell killing through NKG2D ligands and TRAIL receptors [58]. Lee et al.[59] showed in mouse models that resveratrol upregulated NKG2D, NKp30 and CD107a expression, and effectively inhibited tumor growth and metastasis. Stemphol, a natural dialkyl resorcinol extracted from *Stemphylium globuliferum,* induced caspase-independent cell death and released high-mobility group box 1 (HMGB1) in leukemia cells [57]. Daphnetin, a dihydroxylated derivative of coumarin, is a potent stimulator of NK cells in that daphnetin enhances IFN-γ production and direct cytotoxicity in the presence of IL-12 [56]. Daphnetin also suppresses inflammatory cytokine production in experimental autoimmune encephalomyelitis mice [60]. In our study, we showed in ovarian cancer cells that methyl gallate significantly enhanced the expression of stress ligands for DNAM-1 and NKG2D NK cell receptors, i.e. CD112, CD155, MIC-A/B, ULBP-1/2/3, TRAIL-1 (DR4) and TRAIL-2 (DR5) (Fig. 3B, Supplementary Fig. 2B). Pre-treatment of ovarian cancer cells with methyl gallate rendered these cancer cells more susceptible to NK cell killing, compared to ovarian cancer cells that have not been previously exposed to methyl gallate (Figs. 2 and 4). However, gallic acid showed no significant effect on the expression of stress ligands in these cancer cells at the concentration tested (Supplementary Fig. 3), and therefore the relatively low level of NK cell-mediated cytolysis (Fig. 2). In contrast, Dedoussis et al.[61] demonstrated in human leukemia K562 cell line that pre-treatment with 200 µg/ml gallic acid rendered the cells significantly susceptible to NK cell-mediated necrosis. It is likely that the difference in findings could be due to differences in cell type and concentration of gallic acid used. Nevertheless, it is possible that the stress ligands investigated in this study may not account for the whole picture of sensitizing the ovarian cancer cells to NK cell-mediated killing. There may be other ligands and factors not studied here that could potentially contribute to the methyl gallate-associated NK cell lysis or combined methyl gallate and oxaliplatin-associated NK cell lysis, such as B7-H6, calrecticulin, HMGB1, cytokines, and chemokines [46, 47]. It is also unclear whether methyl gallate treatment of ovarian cancer cells inhibited specific signaling pathway, or dampened DNA methyltransferase or histone acetylases. Given the importance of dysregulation of epigenetic signaling pathways and cancer [62], future studies exploring these possibilities are warranted.

As far as we are aware, our group is the first to report the identification and isolation of methyl gallate from leaves of *L. indica* (reference [32] and this study). Also known as methyl-3,4,5-trihydroxybenzoic acid, methyl gallate is a polyphenolic compound reported in plants such as maple leaf [63], root bark of *Paenonia suffruticosa* [64], *Schinus terebinthifolius* [65], *Rosa rugosa* [66], and *Galla rhois* [67]. Methyl gallate has been reported to possess various biological properties including anti-oxidant [64, 68] and anti-microbial properties [69]. In human hepatocellular carcinoma, methyl gallate is reported to suppress cell proliferation via increasing the production of reactive oxygen species and apoptosis [70]. Methyl gallate is also shown to have anti-inflammatory activities in zymosan-induced experimental arthritis animal model, wherein methyl gallate impaired zymosan-stimulated macrophages by inhibiting IL-6 and nitric oxide production, cylooxegenase-2 (COX-2) and inducible nitric oxide synthase (iNOS) expression (71). Administration of methyl gallate in lipopolysaccharide-treated mice protected the mice against acute renal injury, increased anti-oxidant activity and decreased NF-kB activity [64]. In mouse RAW 264.7 cells, methyl gallate blocked inflammation induced by Toll-like receptor ligands through attenuating NF-kB signaling and mitogen-activated protein kinase (MAPK) pathway [72]. Methyl gallate also inhibited lipopolysaccharide-induced nitric oxide and IL-6 production in mouse-derived RAW 264.7 cells, most likely via the down regulation of extracellular-signal-regulated kinase 1/2 (ERK1/2) pathway [73].

Combination of cisplatin-paclitaxel, which is a widely adopted “standard” treatment for advanced ovarian cancer, is frequently interrupted by the emergence of drug resistance cancer cells [74, 75]. Oxaliplatin but not cisplatin was shown to trigger immunogenic cell death of colorectal cancer cells, activated dendritic cells by expressing danger signals such as heat shock proteins, calreticulin, HMGB1, and efficiently generated a pool of tumor antigen-specific T cells [39]. Oxaliplatin is a third-generation platinum compound that is less studied and rarely used but promising in the treatment of ovarian cancer [76]. Oxaliplatin has been the backbone of treatment of colorectal cancer. Its cytotoxic effect is mediated mainly through DNA damage. Like other chemotherapeutic agents when used at high doses, oxaliplatin is reported to have clinically adverse side effects such as peripheral neuropathy and nausea [40–42]. We therefore studied oxaliplatin at low concentration and chose two ovarian cancer cell lines OVCAR-5 and SK-OV-3 with different spectrum of drug resistance. OVCAR-5 cells are known to show resistance to clinically relevant concentrations of adriamycin, melphalan and cisplatin, while SK-OV-3 cells are resistant to tumour necrosis factor and several cytotoxic drugs including diphtheria toxin, cis-platinum and adriamycin. Combination of low level oxaliplatin and methyl gallate in the presence of NK cells was capable of effecting cancer cell lysis despite the tumor resistance (Fig. 4). An E:T ratio of 1:4 was chosen to demonstrate that even at this low concentration of NK cells, the proliferation of these ovarian cancer cells which are resistant to drugs, can still be suppressed (Fig. 5). More importantly, our data suggests that a single treatment regime alone (either NK cell co-culture alone, or methyl gallate treatment alone) was insufficient to completely abrogate cancer cell growth. Likewise, oxaliplatin treatment alone was insufficient [36]. The re-proliferation of minimal residual cancer cells in in vitro cultures was subsequently detectable over time. These “residual” cancer cells can potentially form the next resistant colony in the long term and eventually render resistance to the existing treatment regime. Our work suggests combination of methyl gallate and oxaliplatin, which can trigger antitumor immunogenicity, in conjunction with activated NK cells, warrants further investigation.

The devastating effect of immune dysregulation is well recognized in cancers. Cytokine storm and cytokine release syndrome are life-threatening systemic inflammatory syndromes which involve high levels of circulating cytokines and immune-cell hyperactivation [77]. Multiple studies have demonstrated that ovarian cancer has immunosuppressive tumor micro-environment that poses serious challenge to existing treatment modalities. For instance, myeloid-derived suppressor cells were increased by vascular endothelial growth factor (VEGF) expression in human ovarian cancer, resulting in suppressed immunity [78]. The number of intraepithelial CD8^+^ tumor infiltrating lymphocytes and a high ratio of CD8^+^/Treg are associated with a positive prognosis in epithelial ovarian cancer [79]. Our study showed that methyl gallate inhibited TNF-α and IL-1β production in human U937 macrophages (Fig. 6). Our results are consistent with those findings observed using mouse macrophages [72, 73] in that methyl gallate exert anti-inflammatory effects. In our study, we observed that at the same concentration examined, methyl gallate significantly suppressed TNF-α production (*p* < 0.01), whereas gallic acid showed no appreciable effect on TNF-α production (Fig. 6B). Further, at the same concentration studied, methyl gallate significantly suppressed IL-1β production (*p* < 0.05), while gallic acid had no significant effect on IL-1β production (Fig. 6D). Gallic acid, also known as 3,4,5-trihydroxybenzoic acid, is a natural secondary metabolite and widely present in various plants [80]. Gallic acid is reported to suppress TNF-α and IL-1β levels in gouty arthritis mice model by inhibiting NLR family pyrin domain containing 3 (NLRP3) inflammasome activation [81]. Interestingly, studies on the levels of TNF-α using either RNA in-situ hybridization of tissue arrays or semi-quantitative reverse polymerase chain reaction of mRNA in ovarian cancer have shown that TNF-α expression was present at higher levels in ovarian carcinoma compared to normal tissues [82]. Future studies on understanding the pharmacological mechanism of methyl gallate and its effects on refractory ovarian cancer cells are warranted.

In conclusion, the leaf extracts of *L. indica* and its selected phytoconstituents were successfully investigated for their effects on human ovarian cancer cells and in combination with oxaliplatin and NK cells. The crude leaf extract of *L. indica* was found to enhance the susceptibility of ovarian cancer cells to NK cell cytolysis. Its phytoconstituent methyl gallate was found to upregulate the expression of stress ligands for NK cell receptors and elevate the sensitivity of drug-resistant human ovarian cancer cells to NK cell cytolysis. Our findings suggest that the combined effect of methyl gallate, oxaliplatin and NK cells in ovarian cancer cells warrants further investigation. Our work is a step towards better scientific understanding of the traditional anticancer use of *L. indica*.

## Supplementary Information

**Additional file 1: Supplementary Fig. 1.** Overall flow-chart of the methods. **Supplementary Fig. 2.** Increased expression of stress ligands for NK cell receptors in ovarian cancer cells after treatment with (A) *L. indica* ethyl acetate fraction, (B) methyl gallate, and (C) combination of methyl gallate and oxaliplatin. OVCAR-5 cells were treated for 48 h with or without (A) *L. indica* ethyl acetate fraction (EA, 0.3 mg/mL), (B) methyl gallate (MG, 0.1 mg/mL), or (C) combination of methyl gallate (MG, 0.1 mg/mL) and oxaliplatin (10 µM), and then phenotype analyzed by FACS for the indicated ligands of NK cells: CD112, CD115, MIC-A/B, ULBP-1, ULBP-2, ULBP-3, DR4 (TRAIL-R1), and DR5 (TRAIL-R2). The relative total mean fluorescence intensities (MFI) of each stress ligand were compared between untreated cells (blue solid line) and treated cells (red solid line). The isotype antibody controls are represented by the green dotted line. Numbers indicate the total MFI for each respective ligand. Histograms of one representative experiment of three are shown. **Supplementary Fig. 3.** Gallic acid had no significant effect on the expression of stress ligands for NK cell receptors in human ovarian cancer cells. OVCAR-5 cells were treated with or without gallic acid (0.03 mg/mL) for 48 h and then phenotype analyzed by FACS for the indicated stress ligands of NK cells: (a) CD112, (b) CD115, (c) MIC-A/B, (d) ULBP-1, (e) ULBP-2, (f) ULBP-3, (g) DR4 (TRAIL-R1) and (h) DR5 (TRAIL-R2). The relative mean fluorescence intensities of each stress ligand were compared between untreated cells (blue bars) and treated cells (red bars), and results presented are mean ± SD of three independent experiments. There was no statistical difference between treated and untreated cells for each stress ligand. ns, not significant. **Supplementary Fig. 4.** Schematic figure of some key findings.

## Data Availability

The datasets used and/or analysed during the current study are available from the corresponding authors SYN and HLK on reasonable request.

## Abbreviations

DMSO: Dimethyl sulfoxide
DNAM-1: DNAX accessory molecule-1
ELISA: Enzyme-linked immunosorbent assay
FACS: Fluorescence-activated cell sorting
GC-MS: Gas Chromatography-Mass Spectrometry
HMGB1: High-mobility group box 1
HPLC: High Performance Liquid Chromatography
IC_50_: Inhibitory concentration required for 50% inhibition of activity
IC_20_: Inhibitory concentration required for 20% inhibition of activity
IL-1β: Interleukin-1β
LC-MS: Liquid chromatography-mass spectrometry
LPS: Lipopolysaccharide
MIC-A/B: Major histocompatibility complex class I-related chains A/B
NK: Natural killer
NKG2A: Natural killer, group 2, member A
NKG2D: Natural killer, group 2, member D
PBS: Phosphate buffered saline
PMA: Phorbol-12-myristate-13-acetate
TNF-α: Tumor necrosis factor-α
TRAIL: Tumor necrosis factor-related apoptosis-inducing ligands
ULBP: UL16-binding protein
WST-1: Water soluble tetrazolium salt-1
