# Supplementary material for: Effects of Leea indica leaf extracts and its phytoconstituents on natural killer cell-mediated cytotoxicity in human ovarian cancer
Source: BMC Complement Med Ther. 2023 Mar 11;23:79. doi: 10.1186/s12906-023-03904-1 (PMC10007844; doi:10.1186/s12906-023-03904-1)
Supplement: Supplementary file 1 — Additional file 1: Supplementary Fig. 1. Overall flow-chart of the methods. Supplementary Fig. 2. Increased expression of stress ligands for NK cell receptors in ovarian cancer cells after treatment with (A) L. indica ethyl acetate fraction, (B) methyl gallate, and (C) combination of methyl gallate and oxaliplatin. OVCAR-5 cells were treated for 48 h with or without (A) L. indica ethyl acetate fraction (EA, 0.3 mg/mL), (B) methyl gallate (MG, 0.1 mg/mL), or (C) combination of methyl gallate (MG, 0.1 mg/mL) and oxaliplatin (10 µM), and then phenotype analyzed by FACS for the indicated ligands of NK cells: CD112, CD115, MIC-A/B, ULBP-1, ULBP-2, ULBP-3, DR4 (TRAIL-R1), and DR5 (TRAIL-R2). The relative total mean fluorescence intensities (MFI) of each stress ligand were compared between untreated cells (blue solid line) and treated cells (red solid line). The isotype antibody controls are represented by the green dotted line. Numbers indicate the total MFI for each respective ligand. Histograms of one representative experiment of three are shown. Supplementary Fig. 3. Gallic acid had no significant effect on the expression of stress ligands for NK cell receptors in human ovarian cancer cells. OVCAR-5 cells were treated with or without gallic acid (0.03 mg/mL) for 48 h and then phenotype analyzed by FACS for the indicated stress ligands of NK cells: (a) CD112, (b) CD115, (c) MIC-A/B, (d) ULBP-1, (e) ULBP-2, (f) ULBP-3, (g) DR4 (TRAIL-R1) and (h) DR5 (TRAIL-R2). The relative mean fluorescence intensities of each stress ligand were compared between untreated cells (blue bars) and treated cells (red bars), and results presented are mean ± SD of three independent experiments. There was no statistical difference between treated and untreated cells for each stress ligand. ns, not significant. Supplementary Fig. 4. Schematic figure of some key findings. [file 12906_2023_3904_MOESM1_ESM.pdf]

Supplementary Figure 1.

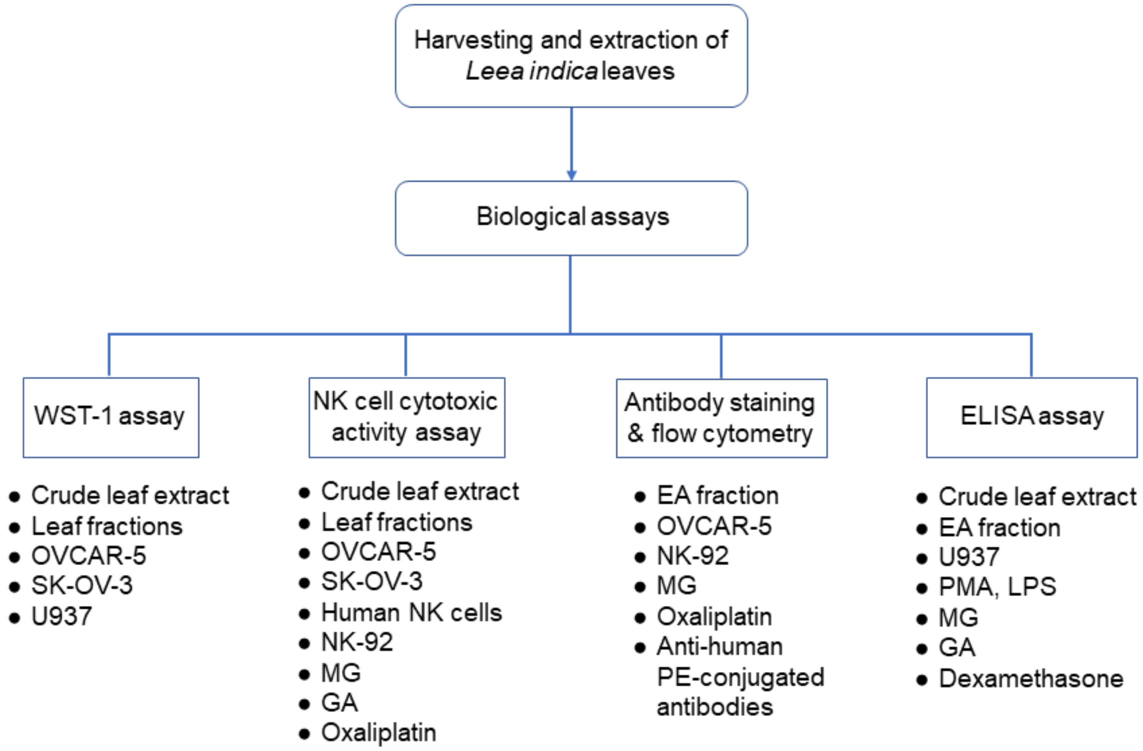

Supplementary Figure 1. Overall flow-chart of the methods.

Supplementary Figure 2.

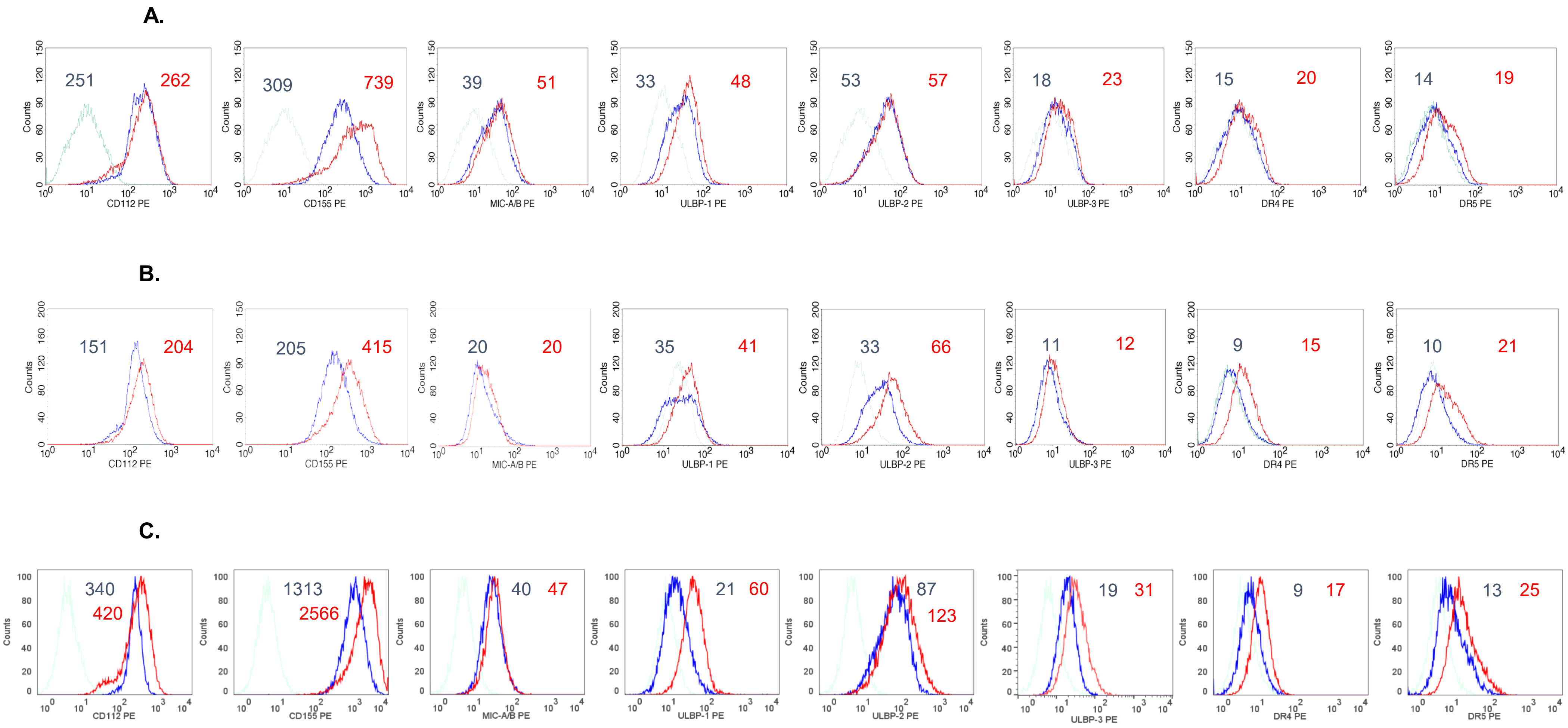

Supplementary Figure 2. Increased expression of stress ligands for NK cell receptors in ovarian cancer cells after treatment with (A) *L. indica* ethyl acetate fraction, (B) methyl gallate, and (C) combination of methyl gallate and oxaliplatin.

OVCAR-5 cells were treated for 48 h with or without (A) *L. indica* ethyl acetate fraction (EA, 0.3 mg/mL), (B) methyl gallate (MG, 0.1 mg/mL), or (C) combination of methyl gallate (MG, 0.1 mg/mL) and oxaliplatin (10 μM), and then phenotype analyzed by FACS for the indicated ligands of NK cells: CD112, CD115, MIC-A/B, ULBP-1, ULBP-2, ULBP-3, DR4 (TRAIL-R1), and DR5 (TRAIL-R2). The relative total mean fluorescence intensities (MFI) of each stress ligand were compared between untreated cells (blue solid line) and treated cells (red solid line). The isotype antibody controls are represented by the green dotted line. Numbers indicate the total MFI for each respective ligand. Histograms of one representative experiment of three are shown.

Supplementary Figure 3.

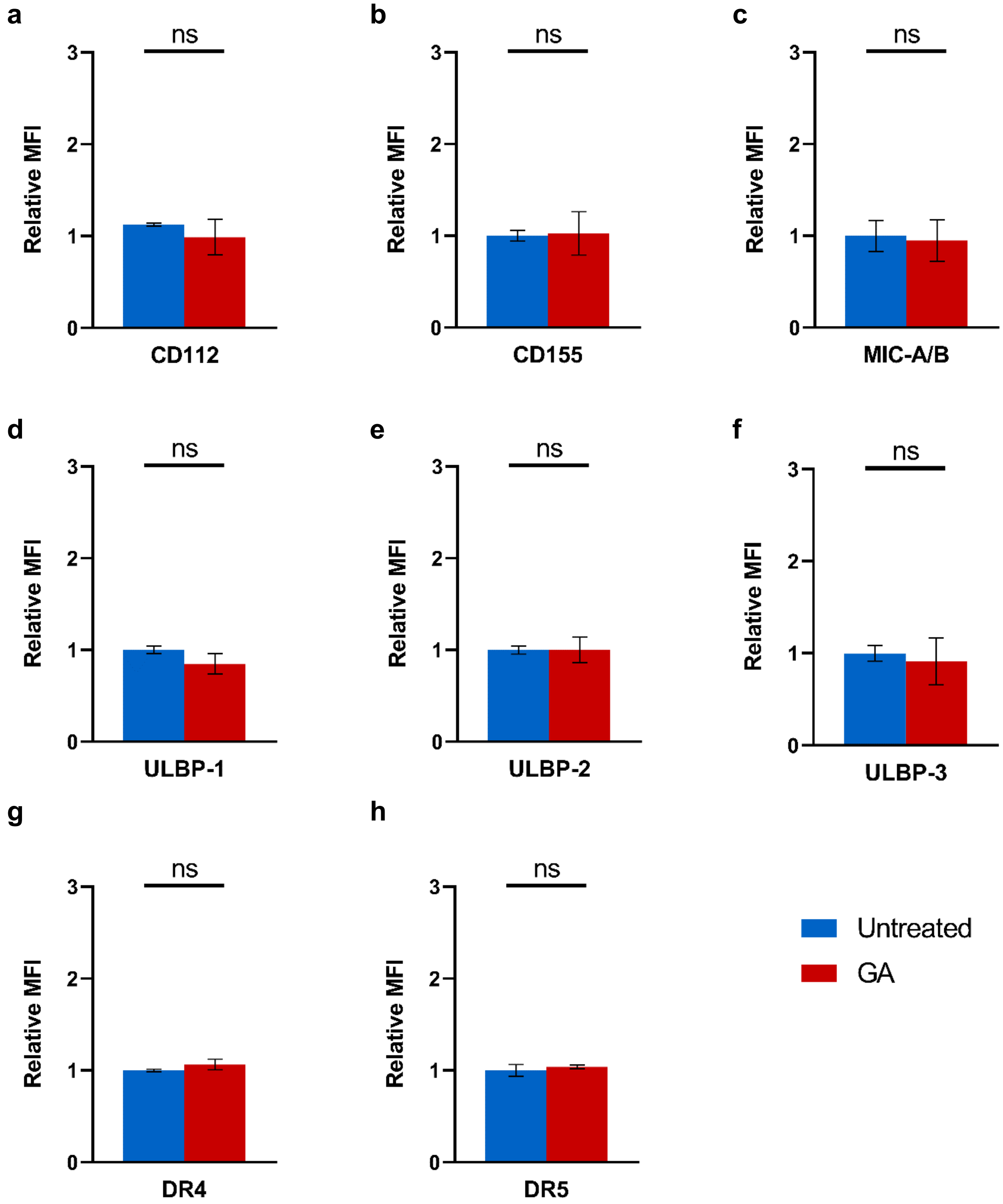

Supplementary Figure 3. Gallic acid had no significant effect on the expression of stress ligands for NK cell receptors in human ovarian cancer cells.

OVCAR-5 cells were treated with or without gallic acid (0.03 mg/mL) for 48 h and then phenotype analyzed by FACS for the indicated stress ligands of NK cells: (a) CD112, (b) CD115, (c) MIC-A/B, (d) ULBP-1, (e) ULBP-2, (f) ULBP-3, (g) DR4 (TRAIL-R1) and (h) DR5 (TRAIL-R2). The relative mean fluorescence intensities of each stress ligand were compared between untreated cells (blue bars) and treated cells (red bars), and results presented are mean ± SD of three independent experiments. There was no statistical difference between treated and untreated cells for each stress ligand. ns, not significant.

Supplementary Figure 4.

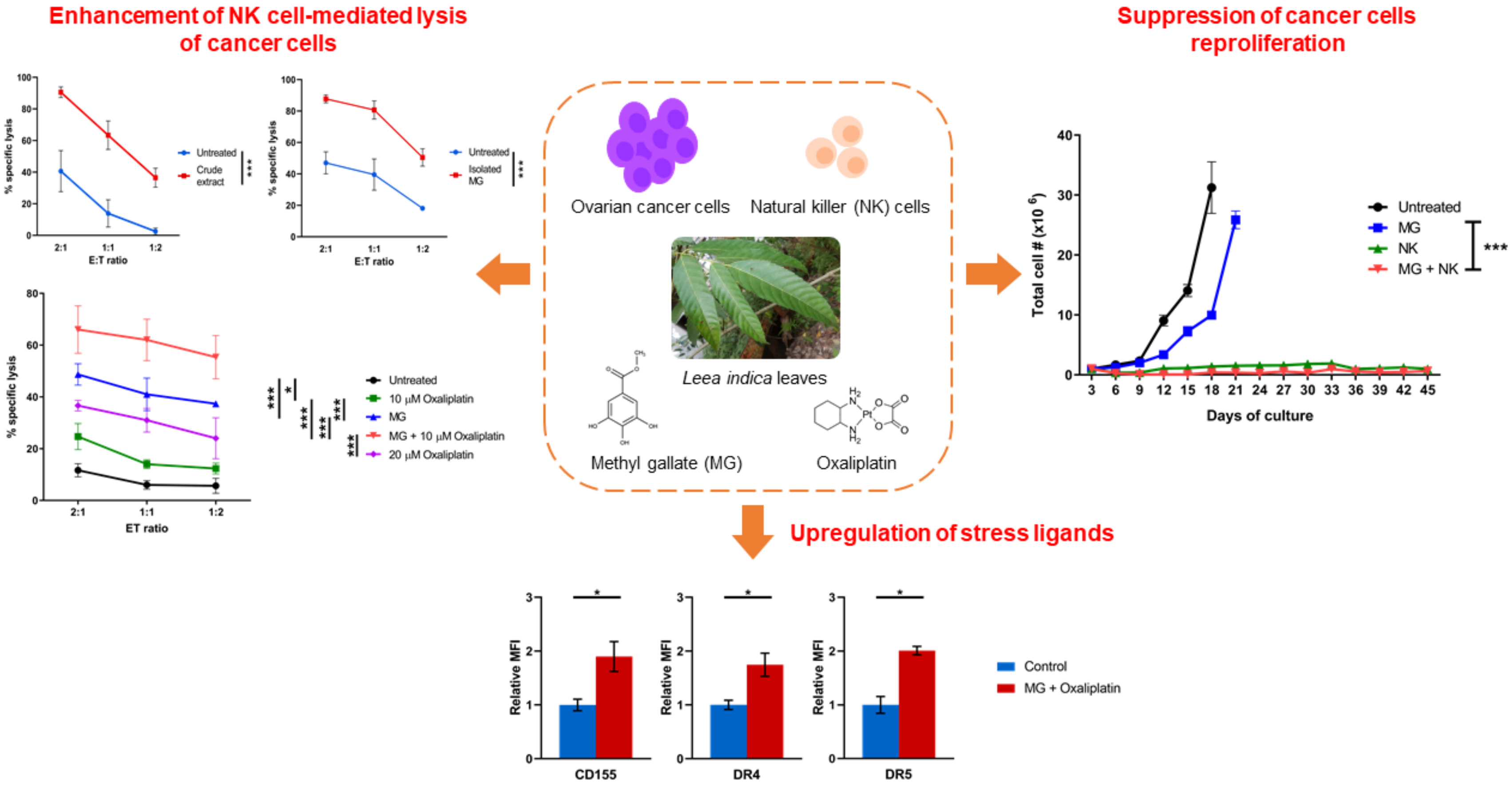

Supplementary Figure 4. Schematic figure of some key findings.
